# Physicochemical Characterization of Interactions between Blueberry Polyphenols and Food Proteins from Dairy and Plant Sources

**DOI:** 10.3390/foods11182846

**Published:** 2022-09-14

**Authors:** Bianca Chima, Paul Mathews, Scott Morgan, Sarah A. Johnson, Charlene B. Van Buiten

**Affiliations:** Department of Food Science and Human Nutrition, College of Health and Human Sciences, Colorado State University, Fort Collins, CO 80523, USA

**Keywords:** polyphenol, protein, digestion, whey, plant-based, protein–polyphenol interaction

## Abstract

Polyphenols are widely known for their benefits to human health; however, dietary intake of this class of compounds is low in the United States due to low intake of fruits and vegetables. Dairy foods (i.e., milk, yogurt) have been shown to increase polyphenol bioavailability via protein–polyphenol interactions, which may have important implications for human health. Increasing consumer interest in sustainability and health has led to the introduction of a variety of novel plant-based proteins and related food products as dairy alternatives. This study compared whey, a popular dairy-based food protein, to pea and hemp proteins for their abilities to form complexes with polyphenols from blueberries, which are a widely consumed fruit in the US with demonstrated health effects. Physical and chemical characteristics of each protein extract in the presence and absence of blueberry polyphenols were investigated using a variety of spectroscopic methods. The influence of polyphenol complexation on protein digestion was also assessed in vitro. While all proteins formed complexes with blueberry polyphenols, the hemp and pea proteins demonstrated greater polyphenol binding affinities than whey, which may be due to observed differences in protein secondary structure. Polyphenol addition did not affect the digestion of any protein studied. Solution pH appeared to play a role in protein–polyphenol complex formation, which suggests that the effects observed in this model food system may differ from food systems designed to mimic other food products, such as plant-based yogurts. This study provides a foundation for exploring the effects of plant-based proteins on phytochemical functionality in complex, “whole food” matrices, and supports the development of plant-based dairy analogs aimed at increasing polyphenol stability and bioavailability.

## 1. Introduction

Polyphenols are a class of secondary metabolites produced by plants and are associated with health benefits to humans upon consumption [1]. Despite these benefits, the dietary intake of polyphenols in the United States is low in comparison to other developed countries due to low intake of fruits and vegetables [2,3]. Presently, berries comprise 10% of the total fruit intake [3] and 7% of the total polyphenol intake of American adults [4]. Recent studies have reported specific health benefits of blueberries, including their usefulness in attenuating cardiovascular disease, type 2 diabetes [5,6], metabolic syndrome [7] and other chronic diseases characterized by oxidative stress and inflammation [8,9,10].

Blueberries are a rich source of dietary polyphenols, comprising between 200 and 400 mg of total phenolics per half-cup serving of fresh fruit depending on factors, such as cultivar and time of harvest [11,12]. The phenolic compounds found in blueberries include phenolic acids, stilbenes, and flavonoids. Chlorogenic acid is the most abundant non-flavonoid found in blueberries, while anthocyanins are the predominant class of flavonoids. Among blueberry anthocyanins, malvidin is found at the highest concentration alongside similar lower concentrations of delphinidin, petunidin, peonidin and cyanidin. Often, these compounds are found in berries in the glycosidic form [11,12,13,14,15,16]. The phenolic compounds found in blueberries are thought to contribute to the health benefits associated with blueberry consumption [8,9,16].

One limitation of the usefulness of polyphenols as a functional food is their poor bioavailability [1,17]; consumption of these compounds does not necessarily translate to their delivery to body tissues. Only approximately 5–10% of ingested polyphenols are absorbed in the small intestine, with the remaining 90–95% continuing to the colon where they are metabolized by the gut microbiota or excreted in feces [18].

Among the factors driving the low bioavailability of polyphenols are chemical instability, degradation in the neutral pH intestinal environment and poor transcellular transport [17]. Through interactions with other molecules in the food matrix, polyphenols can become more or less available for uptake by the intestinal mucosa, a phenomenon described as “bioaccessibility” [19]. Nutrients and ingredients in food products known to influence bioaccessibility and bioavailability include ethanol, fats, emulsifiers, polysaccharides and proteins [20,21,22]. In contrast to modern technologies, such as nanoformulation [22], these nutrient–nutrient interactions take place in all food products regardless of the formulation intention or processing approach.

Interactions between dietary polyphenols with protein from cow’s milk have been studied extensively due to their common combination in food products, such as tea, yogurt, and other blended dairy products. These interactions have been shown to occur spontaneously in food products and result in enhanced polyphenol stability and uptake throughout digestion [23,24,25]. Specifically, the inclusion of milk into formulations has improved the recovery of tea catechins after in vitro digestion [23] and absorption of catechins in an in vitro model of the gastrointestinal tract [24,26].

In recent years, novel proteins have been introduced to the human diet by way of plant-based alternatives to animal-based food products as a response to increasing consumer interest in promoting health, sustainability and animal welfare [27,28,29]. Plant-based dairy alternatives comprise 35% of the total plant-based food market and include products, such as milk, yogurt, ice cream, cheese and protein powders [27]. Recently, legume- and seed-based protein supplements, such as pea and hemp protein, have gained traction with consumers due to decreased concerns about allergens and phytoestrogens in comparison to soy protein [30]. The inclusion of novel plant-based proteins in the diet in place of animal proteins, such as milk or whey protein powder, presents new questions about whether these nutrient–nutrient interactions occur and how these interactions might affect protein digestibility and polyphenol bioavailability.

The objective of this study was to characterize interactions between polyphenols from blueberries (*Vaccinium cyanococcus*) and proteins from novel plant sources (pea, hemp) in comparison to whey protein. The formation of protein–polyphenol complexes was measured at a range of pH levels relevant to food products and human digestion. Binding kinetics were determined using fluorescence quenching, and the influence of protein structure on binding was measured using circular dichroism. Finally, the impact of complexation on protein digestibility was measured in vitro. This information provides a useful foundation for developing plant-based alternatives to common food products with similar physical and chemical properties to their traditional counterparts.

## 2. Materials and Methods

### 2.1. Material Preparation

#### 2.1.1. Blueberry Polyphenol Extraction and Spectrophotometric Characterization

Powdered highbush blueberries (*Vaccinium cyanococcus*) were purchased from Bulk Supplements (Henderson, NV, USA). The blueberry powder was mixed with 50% ethanol at a ratio of 1:5 and adjusted to pH 3.0 with sulfuric acid before mixing for two hours. Ethanol was removed using rotary evaporation at 80 °C. Once cooled, the samples were frozen at −20 °C for further processing.

Samples were diluted 1:1 using acidified water (1% acetic acid in water). C18 solid phase extraction columns (Thermo Scientific, Waltham, MA, USA) were conditioned twice with methanol followed by acidified water. The blueberry extract was added to the column; the column was then washed with three volumes of acidified water. The column was eluted with two volumes of acidified methanol (1% acetic acid in methanol). The solvent was removed from the eluate by rotary evaporation under a vacuum at 80 °C. The resulting blueberry polyphenol extract (BPE) was freeze-dried and stored at −20 °C until use.

Total phenolic content (TPC) of the BPE was determined using the Folin–Ciocalteau assay according to previously established methods [31]. In brief, 10 µL of BPE was added to 790 µL of ultrapure water and 50 µL of Folin–Ciocalteau reagent (Sigma-Aldrich, St. Louis, MO, USA), then shaken for 5 min before the addition of 150 µL sodium carbonate (200 g L^−1^). Samples were incubated for 30 min at 37 °C. After incubation, absorbance was measured at 765 nm using a Bio-Tek UV-Vis plate reader (Winooski, VT, USA). TPC was expressed as gallic acid equivalents based on a standard curve of gallic acid in ultrapure water.

Total anthocyanin concentration (TAC) was measured using the pH differential method as previously described [32,33]. BPE was dissolved at a concentration of 1 mg mL^−1^ in each 0.025 M potassium chloride buffer (pH 1.0) and 0.8 M sodium acetate buffer (pH 4.5). The absorbance of each sample was measured at 500 nm and 700 nm using a V-730 UV-Vis Spectrophotometer (Jasco Inc., Easton, MD, USA). The following equations were used to estimate TAC:

A = (A_500_ − A_700_)_pH 1.0_ − (A_500_ − A_700_)_pH 4.5_ (1)

TAC (µg/mg BPE) = (A × *MW* × 1000)/(*ɛ* × 1) (2)

where *MW* and *ɛ* are the molecular weight and molar absorptivity of cyanidin-3-*O*-glucoside, 449.2 g mol^−1^ and 26,900 L cm^−1^ mol^−1^, respectively.

Total proanthocyanin (PAC) concentration was measured using the 4-dimethylaminocinnamaldehyde (DMAC) method [34,35]. In a 96-well plate, 150 µL of 0.1% DMAC in acidified ethanol was added to 50 µL of BPE (1 mg mL^−1^) or proanthocyanidin A_2_ (PA_2_) standard (1–100 µg mL^−1^) dissolved in 80% ethanol. Absorbance was measured at 640 nm every min for 30 min. PAC concentration of the BPE was expressed as PA_2_ equivalents.

#### 2.1.2. Protein Purification and Quantification

All protein powders were purchased from commercial suppliers (pea protein, Bulk Supplements, Henderson, NV, USA; hemp, Fit Hemp LLC, Melbourne, FL, USA; whey, Nutricost, Vineyard, UT, USA). While the whey protein did not contain fat or carbohydrates, further purification of the pea and hemp proteins was required to remove fat and fiber from the commercial product. Protein powders were defatted with hexane via Soxhlet extraction for 8 h, then underwent alkaline extraction–isoelectric point precipitation to remove insoluble fibers [36]. In brief, the defatted protein powders were dissolved in ultrapure water at a ratio of 1:15 (protein powder:water). The pH was adjusted to 10 for the hemp protein and 9.5 for pea protein using 2 M NaOH. The solutions were then stirred for 2 h at 600 rpm before centrifugation at 4150 rpm for 20 min. The supernatant was collected and adjusted to pH 5.0 (hemp) or pH 4.5 (pea) using 1.2 M HCl before a second centrifugation for 10 min at 4150 rpm. Pellets were collected and resuspended in ultrapure water before adjusting to pH 7.0 with 2 M NaOH. Samples were lyophilized and stored at −80 °C until use.

The protein concentration of each purified sample was determined using a BCA Protein Assay Kit (Thermo-Scientific, Waltham, MA, USA) according to manufacturer instructions. A standard curve was generated using bovine albumin serum (BSA) with concentrations ranging from 0 to 2 mg mL^−1^. Absorbance was measured at 562 nm using a BioTek UV-Vis plate reader (BioTek, Winooski, VT, USA).

### 2.2. Sample Preparation

Samples were prepared to reflect the conditions of a beverage with a single serving of protein supplement (15 g protein) and the polyphenolic equivalent of 175 g of highbush blueberries (1 cup; 490 mg polyphenols) or a concentration ratio of 30:1 (protein:polyphenols) [11]. Samples were prepared to contain whey, pea, or hemp protein in the presence and absence of BPE. The commercial protein powders were used in their unpurified forms in initial turbidity experiments and SDS-PAGE and were prepared to contain 1 mg of whole protein powder per mL and 33 µg of whole blueberry powder per mL. Purified samples had a final concentration of 1 mg protein per mL and when present, 33 µg polyphenols from BPE per mL to achieve a concentration ratio of 30:1. All samples were prepared in 10 mM phosphate buffer at pH 6.8 unless otherwise specified.

### 2.3. Insoluble Complex Formation

#### 2.3.1. Turbidity

The formation of insoluble complexes was assessed by measuring the turbidity of both commercial and purified systems using a V-730 UV-Vis Spectrophotometer (Jasco Inc., Easton, MD, USA). Turbidity was assessed by measuring absorbance at 600 nm. Commercial samples in the presence and absence of whole blueberry powder were measured at pH 6.8. Purified proteins in the presence and absence of BPE were measured at pH 2.0, 4.6, 6.8 and 7.4. These pH levels were selected for relevance to digestion (2.0, gastric; 6.8, duodenal; 7.4 ileac) and food production (4.6, yogurt; 6.8, protein shake).

#### 2.3.2. Sodium Dodecyl Sulfate—Polyacrylamide Gel Electrophoresis

Protein isolates in the presence and absence of BPE were characterized based on molecular weight using sodium dodecyl sulfate–polyacrylamide gel electrophoresis (SDS-PAGE). Samples were prepared as previously described [37]; after mixing, aliquots of each protein-BPE suspension were reserved before the remainder of the sample was centrifuged and the supernatant collected for analysis. Samples were mixed 2:1 with Laemmli sample buffer solution (BioRad Laboratories, Hercules, CA, USA) and electrophoresed on pre-cast tris-glycine gels with 4–20% polyacrylamide (Invitrogen, Waltham, MA, USA) at a constant voltage of 80 V for 10 min followed by 140 V for 60 min. A pre-stained protein ladder of known molecular weights (3.5–250 kDa; Thermo Fisher Scientific, Waltham, MA, USA) was used as a reference for sample protein molecular weights. Gels were stained with Coomassie R-250 dye (Thermo Fisher Scientific, Waltham, MA, USA) and imaged using a UVP EpiChemi3 Darkroom Gel Imaging System (Analytik Jena, Jena, Germany). Images were processed using Image Studio (LI-COR Biosciences, Lincoln, NE, USA).

### 2.4. Circular Dichroism (CD)

A Jasco J-1100 Circular Dichroism Spectrophotometer (Easton, MD, USA) was used to measure the changes in the secondary protein structure of whey, pea, and hemp protein in solution and when bound to BPE. Samples were prepared with a protein concentration of 0.1 mg mL^−1^; polyphenols were added to samples at a ratio of 30:1 (protein:polyphenols). Samples were loaded in a 1 mm quartz cuvette and measurements were taken with the following parameters: wavelength scan from 190 to 360 nm, 50 nm min^−1^; CD scale: 2000 mdeg/1.0 dOD; D.I.T.: 0.5 s; bandwidth: 1.00 nm; data pitch: 2.0 nm; holder temp: 37 °C. Data were processed using JASCO Spectral Analysis software. Blanks and control absorbances were subtracted and spectra were smoothed using the Savitsky–Golay algorithm with a 13-step deconvolution. Changes in relative helicity were measured by dividing the signal of each protein at 222 nm by its signal at 208 nm [38].

### 2.5. Fluorescence Spectroscopy

Fluorescence spectroscopy was used to determine the binding affinities of whey, pea, and hemp proteins for BPE. Protein solutions were prepared at a constant concentration of 0.5 mg mL^−1^ in the presence or absence of BPE. BPE was added to final concentrations of 4–66 µg mL^−1^. Fluorescence intensity (ex: 280 nm; em: 300–450 nm) was measured using an FS5 fluorometer and data wer collected using Flouracle (Edinburgh Instruments, Livingston, UK). The following emission scan parameters were applied: dwell time 0.2, steps: 1 nm, # of scans: 3, and all corrections applied. The fluorescence signal of BPE alone was measured at all test concentrations and subtracted from each corresponding spectrum.

### 2.6. Analysis of Digestibility of Pea, Hemp and Whey Protein with Blueberry Polyphenol Extract

#### 2.6.1. In Vitro Digestion

Whey, pea and hemp proteins underwent in vitro digestion in the presence and absence of BPE according to previously described methods [37]. Samples were prepared at pH 6.8, then decreased to pH 2.0 using 1 M HCl for gastric digestion. Pepsin was added to a final concentration of 0.3 mg mL^−1^ and the samples were incubated at 37 °C in a shaking water bath for 2 h. The pH was then increased to 7.4 using 2 M NaOH and trypsin was added to a final concentration of 0.3 mg mL^−1^, after which all samples were incubated at 37 °C in a shaking water bath for 4 h. At the end of incubation, samples were boiled for 30 min to halt enzymatic reactions.

#### 2.6.2. Determination of Free Amino Acids

The free amino acid content of samples before and after in vitro digestion was measured using the ninhydrin assay [39,40]. Trione ninhydrin reagent (Pickering Laboratories, Mountain View, CA, USA) was combined 1:1 with each sample, which was then heated for 15 min at 95 °C and cooled to 25 °C. Samples were then diluted 1:5 with 50% ethanol. Absorbance was measured at 570 nm using a BioTek UV-Vis Plate Reader. The concentration of free amino acids in each sample was determined using a standard curve of L-leucine (Thermo Fisher, Waltham, MA, USA).

### 2.7. Statistical Analysis

All experiments were performed in triplicate and analyzed using GraphPad Prism v9.3.1 (GraphPad Software, Inc., San Diego, CA, USA). Values are expressed as the mean ± standard deviation. ANOVA was paired with Tukey’s or Šídák’s multiple comparisons to compare samples; values of *p* < 0.05 were considered significant.

## 3. Results and Discussion

### 3.1. Total Phenolic Content and Protein Concentration

The total polyphenol content of the BPE was determined to be 260.1 ± 1.8 mg gallic acid equivalents (GAE) per g extract. The total anthocyanin concentration of BPE was measured to be 21.68 ± 1.7 mg cyanidin-3-glucoside equivalents per g extract. The total proanthocyanidin concentration was measured to be 19.39 ± 1.2 mg PAC A_2_ equivalents per g extract. Each of these values is within the anticipated range based on previously published reports [35,41,42,43].

The protein content of the hemp, pea and whey protein powders were determined to be 73.8%, 50.4% and 69.7%, respectively. The determined values for the pea and hemp protein powders were less than what has been previously reported [36,44,45,46,47], but may be attributed to the use of commercial protein powders as a starting source rather than the raw plant, as well as the method of protein quantitation chosen (i.e., total nitrogen methods versus the BCA assay).

### 3.2. Blueberry Polyphenols Form Insoluble Complexes with Whey, Pea and Hemp Protein in Commercial and Purified Forms

**Figure 1:**
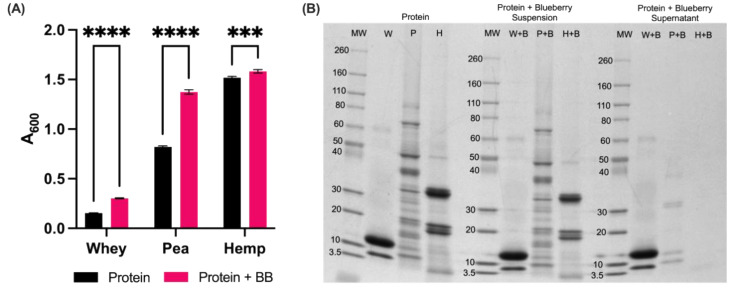
Intermolecular interactions between contents of commercial protein powders and powdered blueberries cause the formation of insoluble complexes. (**A**) Increasing turbidity of commercial protein solutions by blueberry powder points to nutrient interactions. Increases in turbidity exceed absorbance of blueberry-only control (A_600_ = 0.027). Values are expressed as mean ± SD. Asterisks (*) indicate significant differences (*** = *p* ≤ 0.001, **** = *p* ≤ 0.0001) based on two-way ANOVA with Tukey’s multiple comparisons. (**B**) Molecular weight profiles of each commercial protein powder in the absence or presence of powdered blueberries. Molecular weight is represented as kilodaltons (kDa).

The turbidity of commercial protein powder/blueberry powder and purified protein/BPE solutions were measured at pH 6.8. The addition of blueberry powder to commercial protein powders resulted in an increase in turbidity for each protein powder, which suggested that nutrient interactions were causing the formation of aggregates (Figure 1A). Further investigation of these solutions by SDS-PAGE demonstrated that proteins were implicated in the reactions (Figure 1B). Comparison of signal between the proteins alone and the turbid protein powder/blueberry powder suspension demonstrates no difference in signal or molecular weight profiles of the protein and its corresponding protein/blueberry powder molecular weight profile. However, when centrifugation was used to remove insoluble aggregates from the solution, the protein signal decreased, suggesting that proteins were involved in the appearance of turbidity (Figure 1B).

The isolation of proteins and polyphenols from the commercial protein powders and blueberry powder, respectively, allowed for the characterization of interactions occurring specifically between proteins and polyphenols by measuring the turbidity of solutions containing isolated proteins and BPE at 600 nm. Protein–polyphenol interactions can be affected by a variety of parameters, including temperature, pH, protein structure, polyphenol structure, and the relative concentrations of each component of the reaction [48]. The formation of protein–polyphenol complexes is generally driven by non-covalent interactions, such as hydrophobic interaction, hydrogen bonding and van der Waals forces, which are weak yet dynamic, allowing for continued transformation on the basis of changes to a surrounding environment [48,49,50].

**Figure 2:**
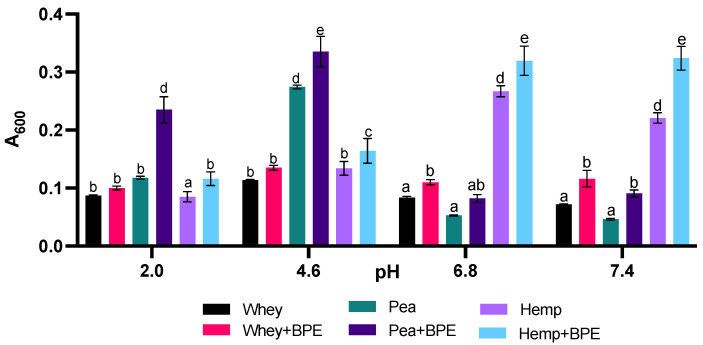
Protein–polyphenol interactions driving increases in turbidity are pH-dependent. Values are expressed as mean ± SD. Different letters indicate significant differences (*p* < 0.05) based on two-way ANOVA with Tukey’s multiple comparisons.

The influence of pH on the physicochemical properties of each protein and its ability to form protein–polyphenol complexes is demonstrated in Figure 2. Increases in turbidity denote a decrease in solubility for a given compound or mixture. Protein solubility is typically at a minimum when the pH of its surrounding environment reaches the isoelectric point of that protein or the pH at which the net charge of the protein is zero. Isoelectric points are unique to specific, isolated proteins, and it is critical to note that the protein isolates from this study comprise more than one protein (Figure 1B), but present literature estimates isoelectric points of 4.5–4.8 for pea proteins, 5.5–6.4 for hemp proteins and 4.5–5.2 for whey protein [46,51,52,53]. Based on these values, the increases in turbidity observed for pea and hemp proteins in the absence of polyphenol-containing blueberry powder can be attributed to pH-induced changes in solubility. Interestingly, whey remains soluble even at its isoelectric point, which explains the relatively small changes in turbidity across pH levels in comparison to pea and hemp protein solutions [52].

The increased turbidity of a solution containing protein and polyphenols has been associated with the formation of protein–polyphenol complexes [37,54,55]. In this study, all proteins demonstrated the ability to interact with BPE and form protein–polyphenol complexes, but these interactions were uniquely affected by pH for each protein source (Figure 2). Notably, pH also seemed to affect the turbidity of the plant proteins in the absence of polyphenols to a greater degree than whey as well. Pea protein showed greater turbidity at pH 2.0 and 4.6 than pH 6.8 and 7.4, while hemp protein showed greater turbidity at pH 6.8 and 7.5 than pH 2.0 and 4.6. Whey, on the other hand, showed only slight increases in turbidity at pH 2.0 and 4.6. While increases in turbidity upon the addition of BPE were observed at all pH levels for the plant-based proteins, for pea and hemp no significant increase in turbidity was observed upon the introduction of BPE to whey protein at pH 2.0 and 4.6.

The overall tendency for turbidity to increase in the presence of BPE supports the hypothesis that each protein tested forms complexes with blueberry polyphenols. The specific differences in turbidity, such as the relative extent to which it is increased at a given pH for an individual protein, suggest that the test proteins behave differently in the presence of polyphenols. These differences could influence their ability to increase polyphenol bioavailability.

### 3.3. Reaction Kinetics Differ Based on Protein Source

**Figure 3:**
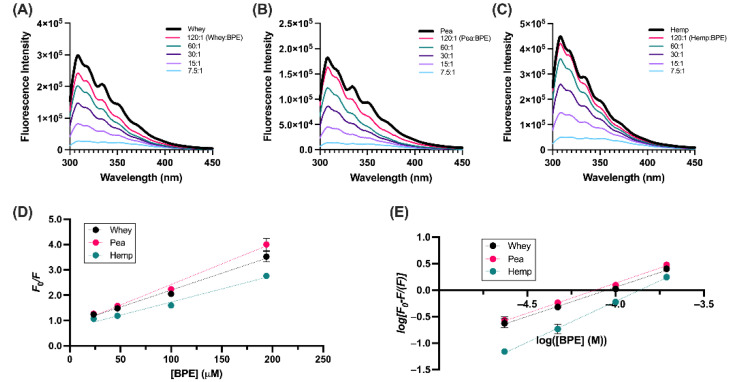
Quenching of protein isolates by BPE. Fluorescence emission spectra (**A**) whey, (**B**) pea and (**C**) hemp as a function of BPE concentration. (**D**) Stern-Volmer plot of F_0_/F as a function of BPE for each protein isolate. (**E**) Double-log Modified Stern-Volmer plot.

Fluorescence spectroscopy was used to determine the binding affinities of each protein to BPE. The fluorescence spectra of each protein showed an emission maximum at ~310 nm after excitation at 280 nm, which can be attributed to the intrinsic fluorescence of tyrosine residues in each protein [56,57,58]. Concentration-dependent decreases in fluorescence intensity occurred upon the addition of BPE to each protein solution (Figure 3A–C), indicating that BPE interacts with the protein, as suggested by the findings from turbidity experiments. Further insights into the kinetics of these interactions were obtained using the Stern–Volmer equation:

*F*_0_/*F* = 1 + *K_SV_*[BPE] = 1 + *k_q_*τ_0_[BPE] (3)

where *F*_0_ and *F* are the fluorescence intensities of the protein in the absence and presence of the quencher, BPE, respectively. The Stern–Volmer quenching constant is defined as *K_SV_*, *k_q_* is the bimolecular quenching constant, and τ_0_ is the average lifetime of the fluorophore of interest in a given solvent.

The quenching of intrinsic fluorophores by ligands, such as BPE, can occur by static quenching, which occurs upon intermolecular interaction, or by dynamic quenching, which occurs upon the collision of two molecules [59]. The formation of protein–polyphenol complexes, demonstrated by UV-Vis (Figure 1 and Figure 2), suggests a static quenching mechanism, which is corroborated by the linearity of the Stern–Volmer plots (Figure 3D). Furthermore, the *K_SV_* of each interaction, determined by calculating the slope of *F_0_/F* versus [BPE] for each protein, was used to calculate the bimolecular quenching constant *k_q_* based on an approximate lifetime of tyrosine fluorescence in water (~3 ns) [56] (Table 1). The comparison of *k_q_* to the maximum possible value of diffusion-limited quenching in water (~10^10^ M^−1^ s^−1^) confirms that the quenching observed is static and a complex is formed between the protein and BPE, as each calculated *k_q_* exceeds this value.

Further information about these interactions can be gleaned by using the following equation:

log[*F*_0_ − *F*/*F*] = log*K* + *n*log[BPE] (4)

where *K* is the apparent binding constant of the protein with BPE and *n* is the number of binding sites; these values can be obtained by plotting log[(*F*_0_ − *F)/F*] versus log[*BPE*] and determining the y-intercept and slope, respectively [60] (Figure 3E, Table 1). Comparison of *K* between proteins suggests that the affinities for interaction with BPE can be ranked hemp > pea > whey.

Differences in polyphenol binding affinity may influence factors related to polyphenol bioavailability, such as uptake of parent polyphenols and the formation of metabolites by the gut microbiome, though a direct relationship between these characteristics has not yet been investigated. It is possible, for example, that the increased affinities for polyphenols measured in the pea and hemp proteins increase polyphenol bioavailability due to increased propensity to interact; conversely, these higher-affinity interactions could have negative implications for bioavailability due to irreversibility, or other anti-nutritive effects, such as the inhibition of protein digestion. As such, the investigation of the biological implications of protein–polyphenol interactions is a critical next step in this line of research.

### 3.4. Circular Dichroism Shows Unique Structural Conformations for Each Protein Source

**Figure 4:**
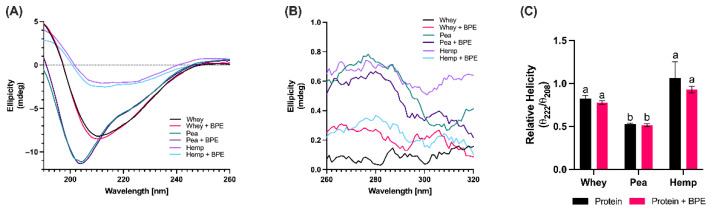
Structural analysis of protein isolates in the presence and absence of BPE at pH 6.8. (**A**) Far-UV spectra of all proteins demonstrate differences in secondary structure between all proteins, but no change upon the addition of BPE. (**B**) Near-UV spectra suggest that all proteins do not have tertiary structure. (**C**) Calculation of relative helicity reveals the helical conformation of whey and hemp, but not pea protein. Helicity is not affected by the addition of BPE. Values are expressed as mean ± SD. Different letters indicate significant differences (*p* < 0.05) based on two-way ANOVA with Tukey’s multiple comparisons.

CD is a spectroscopic technique that is used to characterize protein structure and measure structural transition upon environmental changes. To investigate the influence of protein structure on protein–polyphenol interactions, far-UV CD was used to characterize the secondary structures of pea, hemp, and whey protein isolates (Figure 4A), and near-UV CD was used to evaluate the sidechain environment and tertiary structure of each protein (Figure 4B). These characteristics were also evaluated a second time in the presence of BPE to measure the influence of binding on protein conformation.

The absence of prominent bands in the near-UV (260–320 nm) (Figure 4B) region suggests that all proteins are unfolded and there is no tertiary structure, which is likely due to the manufacturing and purification methods [61]. The far-UV region (190–260 nm) reveals differences in the secondary structures of the protein. Whey and hemp proteins demonstrate structural similarities of predominant α-helices and some β-sheets, as noted by negative bands at 222 nm and 208 nm, and a positive band at 197 nm and 201 nm, respectively [62]. The spectra from pea protein were more consistent with random coil conformation, likely a result of the destruction of the fibrillar structure associated with native, un-denatured pea protein [61].

Polyphenols have been shown to influence protein structure upon interaction in some instances [63,64], but have no or little effect in others [65,66]. The addition of BPE to each protein did not have an effect on structural conformation as evidenced by the absence of differences in each spectrum, as well as the calculation of helicity in the presence and absence of BPE (Figure 4C). These findings are corroborated by the absence of red- or blue-shifting of fluorescence intensity maxima in the presence of BPE as observed in fluorescence spectroscopy (Figure 3A–C).

### 3.5. Physicochemical Properties of Protein Drive Differences in Affinity for Polyphenol Binding

Protein structure, based on both intrinsic and extrinsic factors, plays an important role in the formation of protein–polyphenol complexes. The amino acid composition can heavily influence the propensity of a protein to bind to polyphenols due to specific amino acids’ dielectric properties and physical structure. The amino acid sequence, unique to individual proteins, dictates protein folding, which affects the physical exposure of binding sites to ligands. The findings from UV-Vis and CD experiments suggest that the differences in interaction kinetics are based on differences in protein structure.

Previous studies have characterized the secondary structures of the purified forms of the primary proteins in whey, α-lactoglobulin and β-lactoglobulin. α-Lactoglobulin, which comprises 20% of whey proteins, features 27.6% α-helices, 23.8% β-sheets, 12.6% β-turns and 36.07% random coil, while β-lactoglobulin, which comprises 60% of whey proteins, features 27% α-helices, 29.7% β-sheets, 18.8% β-turns and 24.67% random coil [67]. By contrast, hemp protein isolate has been estimated to comprise 17.4% α-helix, 26% β-sheet, 10% β-turns and 36% random coil [51], while pea protein comprises 8% α-helix, 32% β-sheet, 15% β-turn and 45% random coil [68]. Proteins with compact globular structures typically demonstrate decreased affinity for polyphenols in comparison to loosely structured proteins due to the relative inaccessibility of the peptide backbone [69]. Between pea and hemp proteins, which feature similar frequencies of random coils, it is notable that hemp protein features a greater relative proportion of α-helices and β-turns in comparison to the rigid β-sheets that contribute to the structure of pea protein. Despite the similarities noted between whey and hemp protein in terms of helicity, the relatively low frequency of random coil structure associated with the predominant β-lactoglobulin may drive the differences in binding affinity for polyphenols observed between the two proteins.

Another physicochemical feature that differentiates whey, pea and hemp proteins from one another, and influences binding is molecular weight (MW). As noted in Figure 1B, the MW profiles of each protein differ in terms of both the molecular weight of the predominant species in the sample as well as the range of species. While whey protein features two prominent bands at ~5 kDa and ~15 kDa, representative of α- and β-lactoglobulin, respectively, hemp protein is predominantly detected at ~20 kDa and ~35 kDa, which represent the acidic subunit and basic subunit of the 11S globulin protein [57]. The pea protein isolates produced signals from ~5 kDa to ~80 kDa with varying intensities, likely comprising various vicilins and legumins [36]. These differences in MW profile are relevant because the relative affinities of proteins for polyphenols are influenced by MW, with lower MW peptides demonstrating a lower affinity for polyphenols [69]. The presence of relatively higher MW peptides in both plant-based protein isolates may contribute to the increased binding affinities in comparison to whey protein, and the concentration of proteins with MW > 20 kDa observed for hemp protein may contribute to its greater binding affinity relative to pea protein.

Differences in amino acid profile between each protein in this study may also influence interactions with BPE; as noted previously, each protein source demonstrated pH-dependent differences in solubility (Figure 2), likely based on the unique amino acid profiles and isoelectric points of the proteins in each isolate. The overall proportion of acidic or basic amino acid residence in a protein heavily influences the ability of that protein to participate in non-covalent binding with polyphenols [49]. In the conditions used for both fluorescence spectroscopy and CD, all proteins were above the isoelectric point, meaning that charged amino acids would be deprotonated and hold either a neutral or negative charge. Characterization of the amino acid profiles for each protein source reveals that hemp protein features a greater relative proportion of charged amino acids than pea protein, which features a greater relative proportion of charged amino acids than whey protein [70,71]. This difference in the amino acid profile is a likely contributor to the differences in affinity for interaction with BPE measured by fluorescence spectroscopy.

### 3.6. Polyphenol Complexation Does Not Disrupt Protein Digestion in Model System

**Figure 5:**
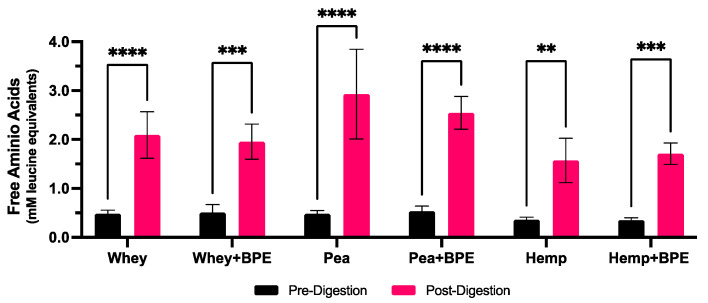
Comparison of free amino acids in solution before and after in vitro digestion in the presence or absence of BPE. Data are expressed as mM leucine equivalents as determined by a standard curve. Values are expressed as mean ± SD. Asterisks (*) indicate significant differences (** = *p* ≤ 0.01, *** = *p* ≤ 0.001, **** = *p* ≤ 0.0001) based on two-way ANOVA with Šídák’s multiple comparisons.

Within the context of food products, the fate of protein–polyphenol complexes is as important as their formation; interactions with proteins have been shown to enhance the gastrointestinal uptake of polyphenols in some cases [19,20,23,72], but prevent the digestion and absorption of proteins in others [37,73]. Thus, the evaluation of the impact of BPE complexation on the digestion of each protein is critical. A comparison of the free amino acid content of the protein isolates before and after digestion in the presence and absence of BPE revealed that complexation did not affect the digestion of any of the proteins (Figure 5).

In each condition, the free amino acid content of the protein increased after in vitro digestion. The pre-digestion free amino acid concentration was approximately 0.5 mM for both the pea and whey protein isolates and 0.35 mM for the hemp protein isolate. After digestion, the free amino acid content increased for each profile isolate between 120 and 130%, regardless of the presence of BPE.

Some studies have shown that protein digestibility decreases significantly when bound to polyphenols; this has been attributed to both the sequestration of the protein being digested as well as the direct inhibition of digestive proteases, such as pepsin and trypsin [37,74,75]. However, this phenomenon is concentration-dependent, and the conditions in which this has been demonstrated feature ratios of polyphenols relative to protein that are much greater than what would be observed in a typical food product. In these cases, free polyphenols are often available to interact with digestive enzymes in greater concentrations than those used in this study by an order of magnitude [37,75,76,77].

In this study, the ratio of 30:1 (protein:polyphenols) is based on estimated concentrations from a protein-rich food product and one cup of blueberries (15 g protein; approx. 490 mg polyphenols) [11]. For this reason, these findings suggest that polyphenol consumption at the levels present in a serving of berries would not impact the digestibility of the proteins which could allow for the proteins to be used as a potential delivery mechanism for the polyphenols.

## 4. Conclusions

Dairy proteins have been noted as effective carriers of polyphenols in mixed food products, but recent consumer trends toward plant-based dairy alternatives demand a better understanding of how plant-based proteins compare to dairy-based proteins in terms of chemical and biological functionality. These findings demonstrate the efficacy of pea and hemp proteins to act as carriers of blueberry polyphenols in a similar manner to whey protein. Hemp protein isolate was shown to have the greatest affinity for interaction with polyphenols followed by pea and whey protein isolates. These differences in affinity are underscored by physicochemical differences between each protein.

These results support further investigation of plant-based proteins as potential delivery systems of polyphenols within the context of food products. An existing body of literature suggests that the complexation of dietary polyphenols can increase bioaccessibility and bioavailability, though these studies feature advanced processing techniques, such as freeze-drying and spray-drying for complex formation [19]. Presently, the influence of the de novo protein–polyphenol complexation that occurs in food products on polyphenol bioavailability is unknown. Future studies are required to understand the influence of these interactions on polyphenol bioavailability, metabolism, and efficacy in terms of conferring the health benefits associated with blueberry polyphenols in the absence of proteins.

## Figures and Tables

**Table 1:** Kinetic properties of protein–polyphenol interactions based on fluorescence quenching.

Protein Source	*K_q_* × 10^12^ (M s^−1^)	*K_SV_* (M^−1^)	*K*	N	r^2^
Whey	4.46 ± 0.20 ^b^	13,375 ± 600.7 ^b^	30,100 ± 1.60 ^c^	1.106 ± 0.048 ^c^	0.9853
Pea	5.40 ± 0.28 ^a^	16,224 ± 849.5 ^a^	47,500 ± 1.44 ^b^	1.136 ± 0.038 ^b^	0.9921
Hemp	3.47 ± 0.23 ^c^	10,372 ± 968.2 ^c^	959,000 ± 1.21 ^a^	1.548 ± 0.053 ^a^	0.9943

*K_q_* = bimolecular quenching constant; *K_SV_* = Stern–Volmer quenching constant; *K* = apparent binding constant for a static quenching model; r^2^ corresponds to the line of best fit for Figure 4E from which *K* and *n* were derived. All values are represented as mean ± standard error. Different letters in the same column denote significant differences between means (*p* < 0.05) based on one-way ANOVA with Tukey’s multiple comparisons.

## Data Availability

Data is contained within the article. The data presented in the figures of this study are available upon request from the corresponding author.
